# Single chamber Mg/Ca analyses of *Globigerinoides ruber* for paleo-proxy calibration using femtosecond LA-ICP-MS

**DOI:** 10.1038/s41597-024-03402-0

**Published:** 2024-06-04

**Authors:** Alexa Fischer, Ralf Schiebel, Klaus Peter Jochum, Lena Heins, Anthea I. Arns, Hedy M. Aardema, Hans Slagter, Maria Ll. Calleja, Noy Levy, Brigitte Stoll, David Walter, Ulrike Weis, Janne Repschläger, Gerald H. Haug

**Affiliations:** 1 Max Planck Institute for Chemistry, Hahn-Meitner-Weg 1, 55128 Mainz, Germany; 2 Institute of Earth Sciences, Ruprechts-Karls-Universität Heidelberg, Im Neuenheimer Feld 234, 69120 Heidelberg, Germany; 3 Department of Earth Sciences, ETH Zurich, Sonneggstrasse 5, 8092 Zurich, Switzerland; 4 Marine Ecology and Systematics, University of the Balearic Islands, Palma de Mallorca, Spain; 5 The Fredy & Nadine Herrmann Institute of Earth Sciences, Hebrew University of Jerusalem, Jerusalem, Israel

**Keywords:** Palaeoceanography, Biogeochemistry

## Abstract

Mg/Ca is an independent proxy in paleoceanography to reconstruct past seawater temperature. Femtosecond Laser Ablation Inductively Coupled Plasma Mass Spectrometry (fs-LA-ICP-MS) was employed to determine the Mg/Ca composition of tests (shells) of the planktic foraminifer species *Globigerinoides ruber albus* (white chromotype) and *G. ruber ruber* (red/pink chromotype) sampled alive from the temperate to subtropical eastern North Atlantic with the research sailing yacht *Eugen Seibold*. Mg/Ca data are compared to (i) the measured *in-situ* temperature of ambient seawater, (ii) average mixed layer temperature, and (iii) sea surface temperature (SST). The pooled mean chamber Mg/Ca from each plankton tow site exhibits a positive relationship with SST. Two chamber-specific calibrations are derived, which are consistent with previous calibration equations for comparable paleo-archives. The results confirm fs-LA-ICP-MS as reliable method for determining Mg/Ca in *G. ruber*, and both the penultimate and antepenultimate chambers of adult specimens may provide comprehensible Mg/Ca temperatures of the surface ocean.

## Background & Summary

Element concentrations and stable isotope ratios in the calcareous shells (tests) of Foraminifera provide information on paleoceanography and past climates^1–3^. The trigonal polymorph of CaCO_3_ allows inclusion of a wide range of trace elements including Li, B, Na, Mg, Mn, Sr, Cd, Ba, Nd, and U^3,4^. Changes in environmental conditions such as temperature and seawater carbonate ion concentration ([CO_3_^2−^]) can produce predictable variations in trace element composition of foraminifer shell calcite^3,5,6^.

Calibrations of ratios between incorporated elements or fractionated isotopes and environmental parameters obtained from foraminiferal calcite provide the basis for paleoclimate reconstruction^7,8^. With the help of culture experiments, plankton tows, sediment traps, and core-top studies, such calibrations were established for several elements^3,9,10^, and the Mg/Ca of planktic foraminifer tests has been established as a proxy of seawater temperature^5,11–16^. Under ideal conditions, these proxies are affected by a single environmental parameter only^12^. Because this is rarely the case, application of these proxies tends to be challenging, and needs to be based on a wide range of empirical data. Since the Mg/Ca is primarily temperature dependent, comparison with δ^18^O from the same tests provides estimates of δ^18^O of the water δ^18^O_sw_, a proxy of ambient seawater temperature and salinity, and global ice volume^17,18^. Here, we provide new high-resolution Mg/Ca data produced with femtosecond Laser Ablation ICP-MS (fs-LA-ICP-MS) on the last three chambers of adult tests of the warm-water planktic foraminifer species *Globigerinoides ruber albus* (syn. *G. ruber*, white) and *G. ruber ruber* (syn. *G. ruber* red/pink), both *sensu stricto* morphotypes, to assess the applicability of data from single chambers as paleo-temperature proxies of seawater of the low-latitude surface oceans in addition to wet-chemical methods applied to entire tests, as proposed by Nürnberg *et al*.^12^.

Foraminifers are major marine archives for climate reconstruction and paleoceanography, owing to the high fossilization potential of their calcareous shell and omnipresence in calcareous sediments of the ocean basins over the past 100 Myrs. The chemical composition of their chambered test is affected by the chemical composition and physical parameters of the environment they live in^1^. These marine protozoans live in the surface to sub-thermocline layer of the open ocean and deep marginal seas^1^. Their shell usually consists of low-Mg calcite layers orientated in a radial structure (Arns *et al*., 2022, and references therein)^19^.

The temperature dependence of the partitioning of Mg into ontogenetic foraminifer calcite is the basic principle of Mg/Ca palaeothermometry^20^. Mg/Ca is affected by temperature and other factors such as salinity, *p*H, and ∆CO_3_^2−^ ^12,21,22^. Mg/Ca tends to increase with increasing salinity^12,21^ and decreasing *p*H^3,21^. In addition, alternating high and low Mg/Ca bands within the chamber walls of single specimens are assumed to result from diurnal changes in Mg uptake^23,24^.

Traditionally, Mg/Ca thermometry relies on the bulk analysis of samples comprising 10–30 foraminifer tests of the same species providing averaged temperature signals incorporated at different habitat depths^5,14,16,25^. Recently new techniques such as LA-ICP-MS were developed and facilitate investigation of the underlying mechanism of differences in Mg/Ca composition by profiling Mg/Ca distribution across individual chambers of the same test over a month-long (on average) life cycle^26–28^.

In this study, fs-LA-ICP-MS was employed to determine and compare the Mg/Ca composition of different chambers of the spinose, symbiont-bearing planktic foraminifer *G. ruber* at high resolution across the walls of the last three chambers (final, F0; penultimate, F-1 (final minus one); and antepenultimate, F-2) of individual foraminifer tests. Due to the shallow dwelling depth, *G. ruber* constitutes an excellent archive for studying past hydrological changes in tropical and subtropical surface oceans^1,5,15^. *Globigerinoides ruber* were collected onboard *S/Y Eugen Seibold* during three research cruises in 2019 in the Madeira Basin. Finally, new chamber-specific equations for the relationship between Mg/Ca and ambient water temperature are presented and compared to existing Mg/Ca-temperature equations.

## Methods

### Sampling and sample processing

Samples were collected in the subtropical eastern North Atlantic with the *S/Y Eugen Seibold* during cruises ES19C08, ES19C12, and ES19C14 in 2019 (Table 1) with a bongonet and a multinet (both Hydrobios^®^, Germany) from different water depth intervals (Table 2). The samples were preserved in a hexamine buffered 3% formalin solution at *p*H > 8.2 immediately after sampling. Ambient temperature was measured using Temperature-Depth (TD48, Sea&Sun Technology^®^, Germany) and Conductivity-Temperature-Depth (CTD75M, Sea&Sun Technology^®^, Germany) probes attached to the bongonet and multinet, respectively (Table 2).

**Table 1:** Scientific expeditions of *S/Y Eugen Seibold* in 2019.

Cruise Number	Year	Month	Start Day End Day	Start Time End Time	Start Port End Port	CSS	Latitude Longitude	Water Depth (m)	Abbreviation CSS
ES19C08	2019	5	8	12:00 18:00	Las Palmas, Gran Canaria, ES Marina Rubicon, Lanzarote, ES	8	29.5066 −15.0045	3560	ESTOC*
ES19C12	2019	6	20–21	06:00 19:00	Quinta do Lorde, Madeira, PT Ponta Delgada, Azores, PT	12	32.9862 −22.0469	5350	33 N 22 W
ES19C14	2019	8	13–14	07:00 17:00	Ponta Delgada, Azores, PT Vigo, ES	15	40.0003 −20.0009	4800	40 N 20 W

7-digit campaign labels give Eugen Seibold (ES) Year Year (19) Cruise (C), and Digit1 Digit2 (D1D2) for the consecutive expedition number, reading ESyyCD1D2. Time is given as UTC. CSS is Central Sampling Site. Negative longitudes indicate degrees western hemisphere. Latitudes and longitudes give the targeted sampling sites, and actual samples were obtained adrift within the same water body, and over some distance from the starting point. PT is Portugal, ES is Spain, ESTOC* is near the European Station for Time-Series in the Ocean Canary Islands (ESTOC).

**Table 2:** Mg/Ca of *Globigerinoides ruber*. F0, F-1, and F-2 are the final, penultimate, and antepenultimate chambers, respectively.

Sample Number	Device	Water Depth (m)	F0	1 σ F0	F-1	1σ F-1	F-2	1σ F-2	SST	T MLD	T *in-situ*
ES19C08_008_01	MN	20–40	1.48	0.10	2.69	0.23	2.08	0.24	19.75	19.70	19.64
ES19C08_008_01	MN	40–60	2.43	0.21	3.55	0.19	3.20	0.15	19.75	19.70	19.50
ES19C08_008_01	MN	20–100	2.13	0.18	3.23	0.25	3.01	0.24	19.75	19.70	19.50
ES19C08_008_02	MN	150–500	2.83	0.23	3.66	0.25	3.51	0.27	19.66	19.66	n.app.
ES19C12_012_03	BN	50	2.53	0.28	3.89	0.25	4.58	0.40	21.91	21.62	21.20
ES19C12_012_06	BN	50	3.88	0.22	3.86	0.38	4.71	0.44	22.31	21.84	21.08
ES19C12_012_05	MN	60–100	n.a.	n.a.	3.87	0.24	4.70	0.36	22.44	22.03	n.a.
ES19C12_012_10	MN	0–20	5.00	0.63	5.11	0.21	3.72	0.25	22.04	21.59	22.38
ES19C12_012_10	MN	60–100	2.45	0.25	4.31	0.24	3.74	0.26	22.04	21.59	n.a.
ES19C14_015_03	BN	40	2.08	0.24	3.84	0.20	4.08	0.30	20.45	19.37	17.32

SST is sea surface temperature, T MLD is average mixed layer temperature, and T *in-situ* is ambient temperature per sampled water depth interval. Temperatures are in degrees Celsius (°C). n.a. is not available, n.app. is not applicable because settling foraminifer tests were sampled from the subsurface water column below their live habitat. For metadata on the sampling locations and sample numbers see Table 1. MN is multinet, BN is bongonet. For primary data see files MgCa_avg.tab and MgCa_per_laser_spot.tab in 10.17617/3.D9FSSN^35^.

In the micropaleontology laboratory at the Max Planck Institute for Chemistry (MPIC, Mainz), the plankton net samples were rinsed with one liter of tap water. The planktic foraminifers were picked from the solution with a glass pipette, dried at room temperature, and identified at the species level according to Schiebel and Hemleben (2017). For Mg/Ca analyses, 21 clean individuals of the planktic foraminifer *G. ruber* were selected from the >100 μm size fraction. Additional chemical (reductive and oxidative) cleaning procedures were not applied to the tests to not alter the original and pristine shell material. The foraminifer tests were glued (Tylopur MOBS 4000, ShinEtsu, 1:100 additive-free methylhydroxypropylcellulose (MHPC), Wiesbaden, Germany) onto a glass slide with the spiral side, with the umbilical side facing up and exposing the final three chambers for LA-ICP-MS analyses^29^.

### Laser ablation, mass spectrometry, and Mg/Ca temperature calculation

Magnesium and calcium of 19 specimens of *G. ruber albus* and two *G. ruber ruber* tests were measured at high-resolution on either two or three chambers of each test using a 200 nm wavelength NWR femtosecond laser ablation system (NWRFemto) from Electro Scientific Industries (ESI, New Wave Research Division, Portland, USA) combined with a ThermoFinnigan high-resolution sector-field ICP-MS Element2 mass spectrometer^27,30,31^.

Femtosecond LA-ICP-MS (fs-LA-ICP-MS) analyses were performed on 45 μm diameter laser spots on each chamber with a pulse repetition rate (PRR) of 1 Hz at low fluence of 0.1 J/cm^2^ ^27^. MACS-3 was used as reference material for calibration at the beginning of analyses of the objects and every 2 hours in the following, i.e., after each 27 to 30 measurements of the analyzed objects^32^. MACS-3 is a homogeneous pressed powder pellet consisting of synthetic calcium carbonate powder provided by the United States Geological Survey (USGS), used for calibration with the mass fractions of Mg = 1756 μg/g and Ca = 37.69% m/m^33^.

The ablated material was analyzed with a single collector sector-field SF mass spectrometer (ThermoFinnigan Element2), with electric and magnetic fields, operated at low mass resolution mode. Employing fast (<0.001 s) electric jump, the double-charged ^44^Ca^2+^, which is within a similar mass range (m/2e = 22) as ^25^Mg^+^ (m/e = 25) was measured^27^. This adjustment shortens measurement time and improves the measurement precision. The disadvantage of this technique is the difference between the masses of interest, which must not be more than 30% apart^27^. Accordingly, ^44^Ca and ^25^Mg measurements were carried out on the less abundant double-charged ^44^Ca rather than on the single charged Ca ions^27^.

Raw fs-LA-ICP-MS data were evaluated with an automated Microsoft Excel application, at a minimum of ^44^Ca^2+^ and ^25^Mg^+^ count rates per second^27^. The washout time of the ESI Large Format Cell is one second, thus the individual peaks were separated due to their shorter lengths of about 0.9 seconds. Data calibration was conducted on the MACS-3 reference material during each session. Obvious outliers and unusually high trace element abundances at the beginning of the ablation (1–3 seconds) were identified and rejected by the Microsoft Excel routine aiming at excluding data from potential surface contamination^27,34^. Ablation profiles (see Supplement) were optically inspected for verification of the different ablation intervals and to only interpret data from the ontogenetic calcite. In order to correct for interferences, background data were subtracted from the individual ion intensities. The background-corrected count rates of ^25^Mg were divided by the background-corrected count rates of ^44^Ca for each scan^34^. The trace element concentration C_El_ (μg g^−1^) is given by

1 $${{\rm{C}}}_{{\rm{El}}}={{\rm{C}}}_{{\rm{El,uncorr}}}\ast 1/{\rm{RSF}}$$

with C_El,uncorr_ being the apparent (uncorrected) concentration, and RSF being the relative sensitivity factor^34^. C_El,uncorr_ is determined by:

2 $${{\rm{C}}}_{{\rm{El,uncorr}}}={{\rm{C}}}_{{\rm{IS}}}\ast {{\rm{R}}}_{{\rm{ik}}}\ast {{\rm{A}}}_{{\rm{k}}}/{{\rm{A}}}_{{\rm{i}}}\ast {{\rm{M}}}_{{\rm{EL}}}/{{\rm{M}}}_{{\rm{IS}}}$$

with C_IS_ being the concentration (μg g^−1^) of an internal standard element (IS), R_ik_ being the ratio of the ion intensities of the isotope i of the element of interest (EL), and of isotope k of the internal standard element. A_i_ and A_k_ are the isotopic abundances of isotope i and k, respectively^34^. M_EL_ and M_IS_ are the relative atomic masses of the element of interest and the internal standard element, respectively^34^. The RSF is determined by:

3 $${\rm{RSF}}={{\rm{C}}}_{{\rm{El,uncorr}}}/{{\rm{C}}}_{{\rm{El,true}}}$$

with C_El,uncorr_ being the uncorrected concentration of the element El obtained by reference to the internal standard element, and C_El,true_ being the ‘true’ concentration in a reference material^34^, with values (mass fractions in mg kg^−1^) for MACS-3 of Mg = 1756, and Ca = 376900^33^.

The measurement precision (1 relative standard deviation in percent, 1RSD, 1 s) of the Mg/Ca, is determined by repeated measurements of homogeneous calcium carbonate reference materials. Six to 12 independent analyses of the pressed power pellets of MACS-3 yield uncertainties (1 RSD) in Mg/Ca between 4.96% and 7.34%.

The 3-standard deviation and the mean of the blank estimates the limit of detection (LOD)^27^. For calcareous samples, such as foraminifer tests, the Ca content is high and uniform at about 40% m/m^27^. Therefore, the LOD of Mg/Ca mainly depends on the LOD of Mg ranging at 0.4–1.2 mg kg^−1^ ^23^.

To test data reliability, Mg/Ca values were converted into ambient water temperature estimates according to

4 $${\rm{Mg}}/{\rm{Ca}}={be}^{{\rm{(}}aT{\rm{)}}}$$

with a = 0.38 and b = 0.09 for *G. ruber*^5,15^.

## Data Records

The raw (MgCa_per_laser_spot.tab) and averaged (MgCa_avg.tab) Mg/Ca data are available at 10.17617/3.D9FSSN^35^ on the Edmond Open Research Data Repository of the Max Planck Society, Germany. All column headings of the metadata (sample ID, latitude, longitude, time of sampling), measured Mg/Ca data, and statistical data, i.e., 1σ (1RSD, ‘Error’) are detailed in the headers of the data files. Temperature (°C) of ambient seawater at the time of sampling (also Table 2, T *in-situ*) is given for comparison with the calculated temperature of the measured Mg/Ca of the shell carbonate of the respective planktic foraminifer species *G. ruber* (white) or *G. ruber* (red).

Sample identification (ID) in the data tables numbers provide information per expedition of the sailing yacht *Eugen Seibold* (ES), and year of sampling (e.g., ES19 in 2019). Cruise numbers are consecutive and start with cruise number one each year (e.g., *C08* for cruise ES19*C08*). The cruise number is followed by the number for station, cast, and sample with a consistent format. Station numbers have three digits (e.g., 008 in ES19C08_*008)*. Cast labels have two digits (e.g., 01 as in ES19C08_008_01). Sample labels have one digit (e.g., 1 as in ES19C08_008_01_1). Hence, the label ES19C08_008_01_1 identifies a sample taken onboard the *S/Y Eugen Seibold*, in 2019, on Cruise 8, at Station 8 in the same year, obtained with the first cast at station, and subsampled from the first multinet employed at the same station (Table 2).

The hydrological conditions including ambient seawater temperature have been determined from CTD48 and CTD75M data (Table 2) gathered during cruises ES19C08, ES19C12, and ES19C14 (Tables 1, 2).

## Technical Validation

### Relationship of Mg/Ca data between the different chamber F0, F-1, and F-2 of *G. ruber*

**Fig. 1:**
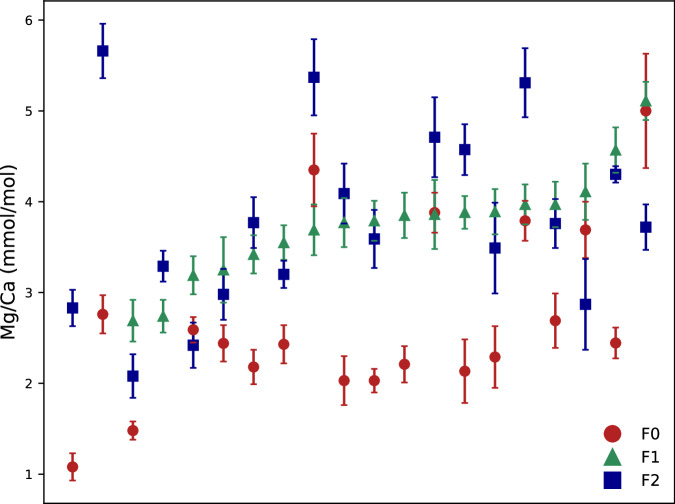
Mg/Ca data of planktic foraminifer shell calcite from fs-LA-ICP-MS analyses. Comparison of the Mg/Ca values obtained for different chambers in 20 *G. ruber* tests. The final chamber (F0), penultimate chamber (F-1), and antepenultimate chamber (F-2) are shown by red dots, green triangles, and blue squares, respectively. Data sorted according to Mg/Ca in F-1. Error bars are in 1σ.

The majority of Mg/Ca data (MgCa_per_laser_spot.tab) range at 1–6 mmol/mol and are considered reasonable (Fig. 1). The high Mg/Ca values above 20 mmol/mol of the three chambers of one *G. ruber* (pink) individual from ES19C12_012_06 (Table 2) are considered not realistic and may not be used for further analyses. F-test and t-test show that the remaining Mg/Ca data from the F-1 ($\overline{x}$ = 3.74 ± 0.58, n = 16) and F-2 ($\overline{x}$ = 3.79 ± 1.00, n = 17) chambers are statistically similar, and significantly different from F0 ($\overline{x}$ = 2.71 ± 0.99, n = 17).

### Relationship between single chamber-Mg/Ca values of *G. ruber* and measured water temperature

**Fig. 2:**
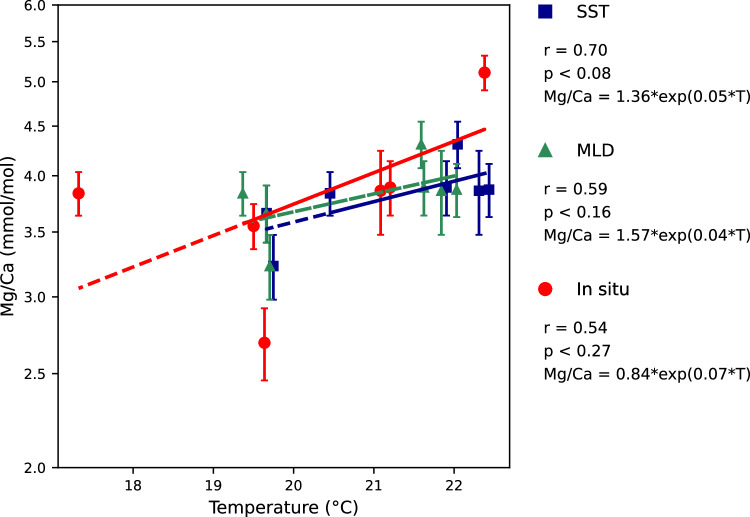
Mg/Ca of the penultimate chamber (F-1) calcite of *G. ruber* per measured temperature. Relationship between measured Mg/Ca (ln scale) of the penultimate chamber (F-1) and SST, over the surface mixed layer (MLD, mixed layer depth), and *in-situ* temperatures. Error bars are in 1*σ*. Sensitivities of Mg/Ca *vs*. SST, average surface mixed layer temperature, and *in-situ* temperature are 5 ± 2%, 4 ± 3%, and 7 ± 5% per 1 °C, respectively.

**Fig. 3:**
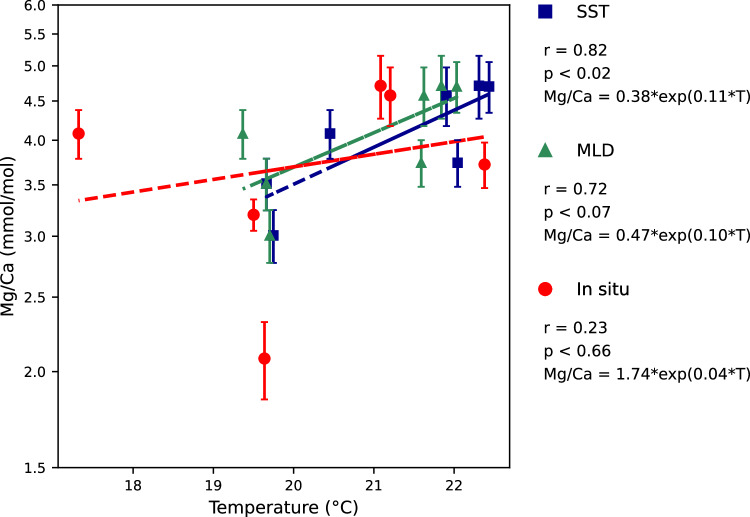
Mg/Ca of the antepenultimate chamber (F-2) calcite of *G. ruber* per measured temperature. Relationship between measured Mg/Ca (ln scale) of the antepenultimate chamber (F-2) and SST, surface mixed layer (MLD, mixed layer depth), and *in-situ* temperatures. Error bars are in 1σ. Sensitivities of Mg/Ca *vs*. SST, average surface mixed layer temperature, and *in-situ* temperature are 11 ± 4%, 10 ± 4%, and 4 ± 7% per 1 °C, respectively.

The Mg/Ca of the penultimate (F-1; Fig. 2) and antepenultimate chambers (F-2; Fig. 3) of *G. ruber* increases with the SST, temperature of the surface mixed layer, and *in-situ* temperature. The Mg/Ca of the penultimate (F-1) and antepenultimate chambers (F-2) show statistically significant relationships with SST (Figs. 2, 3). In comparison, the relationships of Mg/Ca of both penultimate (F-1) and antepenultimate (F-2) chambers with the mixed layer temperatures are rather weak (Figs. 2, 3). The Mg/Ca relationships to *in-situ* temperatures are weakest among the three different temperature measures (Figs. 2, 3).

The relatively poor numerical relationship of the Mg/Ca of the antepenultimate chamber (F-2) with the *in-situ* temperature may be explained by the temporal distance of chamber formation and sampling date of the foraminifer shell in the respective water body, i.e., typically a couple of days to weeks between the formation of the F-2 and the final chamber^36–38^. The question remains, why -on average- SST does best explain the Mg/Ca of the penultimate and antepenultimate chambers (Figs. 2, 3). The fact that the Mg/Ca of final chamber (F0) shows weak relationships with any measure of seawater temperature recorded at the time of sampling may be explained by the potentially incomplete calcification of the chamber wall^1,39^.

### Relationship between single chamber-Mg/Ca temperature of *G. ruber* and measured water temperature

Mean Mg/Ca from each chamber of the analyzed *G. ruber* (Table 2) are used to assess the relationship with the sea surface temperature (SST), ambient water temperature (*in-situ*), and average mixed layer temperature, ranging from the sea surface to the upper limit of the thermocline, by applying the relationship formulated by Dekens *et al*.^15^ and Anand *et al*.^5^ (Table 2).

**Fig. 4:**
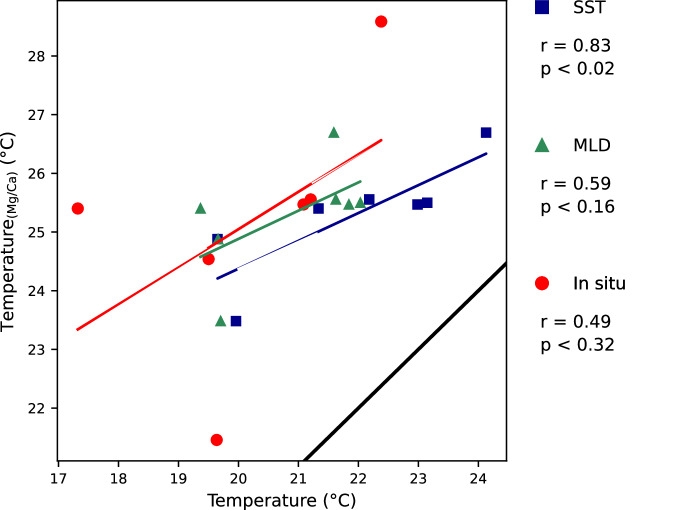
Calculated Mg/Ca temperatures of the penultimate chamber (F-1) *G. ruber* per measured temperatures. Comparison of calculated Mg/Ca temperature (°C) after Dekens *et al*.^15^ and Anand *et al*.^5^ versus measured temperatures, SST, surface mixed layer (MLD), and *in-situ*. Black line marks 1:1 relationship.

Comparing the calculated temperatures (°C) of penultimate chamber (F-1) to the measured SST a statistically significant correlation is revealed at r = 0.83, and p < 0.02 (Fig. 4). With the average mixed layer temperature and *in-situ* temperature being statistically not significant, only positive trends can be detected with r = 0.59 and r = 0.49, respectively.

**Fig. 5:**
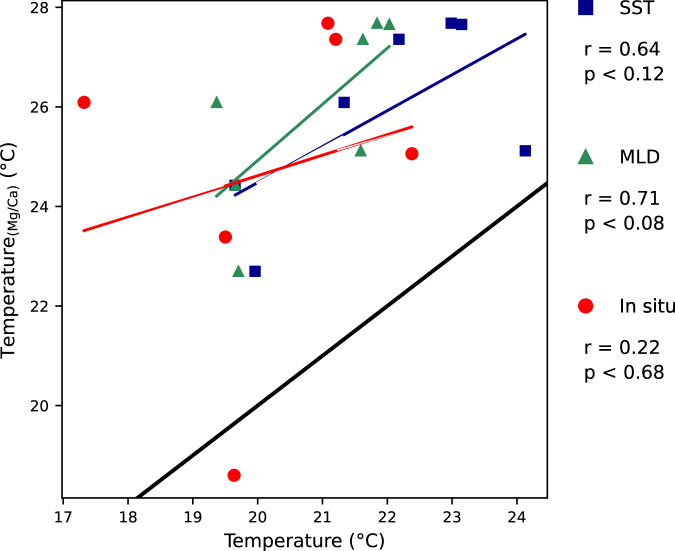
Calculated Mg/Ca temperatures of the antepenultimate chamber (F-2) *G. ruber* per measured temperatures. Comparison of calculated Mg/Ca temperature (°C) after Dekens *et al*.^15^ and Anand *et al*.^5^ versus measured temperatures, SST, surface mixed layer (MLD), and *in-situ*. Black line marks 1:1 relationship.

Comparing the calculated temperatures (°C) of antepenultimate chamber (F-2) to the measured temperatures, no statistically significant relationship exists with SST (r = 0.64) and average mixed layer temperature (r = 0.71). Calculated Mg/Ca temperatures show no relationship (r = 0.22) or trends *in-situ* temperatures (Fig. 5).

**Fig. 6:**
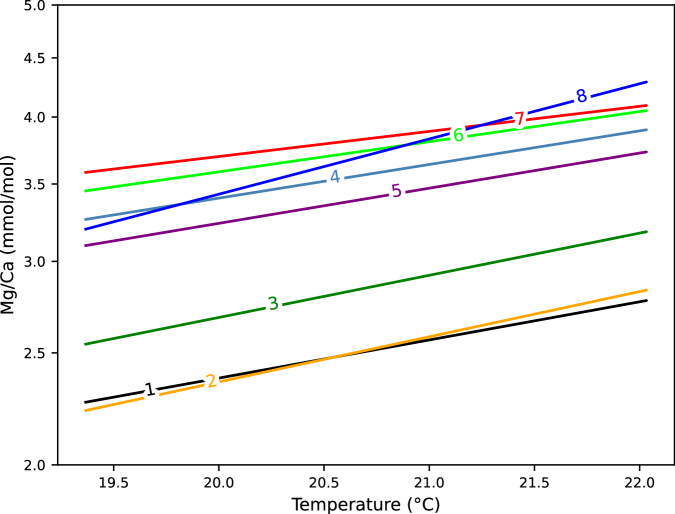
Paleothermometers developed in the present study compared to other Mg/Ca-paleothermometers. Two thermometers (Lines 7 and 8) derived here from Mg/Ca data of the penultimate chamber and SST data (7) as well as from the antepenultimate chamber (F-2) and mixed layer temperature data (8) of *G. ruber*, compared to the findings of (1) Sadekov *et al*. (2009; core top samples, LA-ICP-MS)^26^, (2) Dekens *et al*. (2002; core top samples, wet chemistry)^15^ and Anand *et al*. (2003; sediment trap samples, wet chemistry)^5^, (3) Mohtadi *et al*. (2009; sediment trap samples, wet chemistry)^40^, (4) Bolton *et al*. (2011; LA-ICP-MS, F-1)^39^, (5) Bolton *et al*. (2011; LA-ICP-MS, F-2)^39^, and (6) Gray *et al*. (2018; sediment trap samples, wet chemistry)^21^.

Finally, the Mg/Ca temperatures produced here range at the upper limit of the Mg/Ca temperature of *G. ruber* of earlier analyses, which have analyzed various types of samples and species with different methodologies (Fig. 6, and references therein). In particular, absolute values and slope of the regression of the data from the penultimate chambers (Fig. 6, Line 7) are close to the values of Bolton *et al*. (2011; LA-ICP-MS of single chambers)^39^, and the salinity and *p*H corrected values of Gray *et al*.^21^, whereas the slope of the regression of the data from the antepenultimate chambers (Fig. 6, Line 8) is steeper than in Gray *et al*.^21^. The high-resolution data set provided here is based on a limited number of individual *G. ruber* representative of the 19–22 °C temperature range, and may be used together with other data of similar nature and quality for a broader application of the Mg/Ca thermometer at the basin to global scale.

### Supplementary information

Fischer et al Supplement MgCa profiles

## Data Availability

Custom code was not used in this study.
