# Supplementary material for: Single chamber Mg/Ca analyses of Globigerinoides ruber for paleo-proxy calibration using femtosecond LA-ICP-MS
Source: Sci Data. 2024 Jun 4;11:583. doi: 10.1038/s41597-024-03402-0 (PMC11150449; doi:10.1038/s41597-024-03402-0)
Supplement: Supplementary file 1 — Fischer et al Supplement MgCa profiles [file 41597_2024_3402_MOESM1_ESM.pdf]

Fischer et al., Supplement. fs-LA-ICP-MS Mg/Ca ablation data (1 Hz) per ablation time in seconds. Data used for Mg/Ca temperature calculation are from the inside of the test wall and shaded in grey (interval is also given below the graph). The signal from the outside of the shell wall (left side of graphs) may include contamination from cytoplasm or other calcite, which is not ontogenetic. Data from the inside of the test wall and deep ablation crater (right side of graphs) may vary because of methodological reasons, or because the ontogenetic test wall was entirely ablated, and excluded from further analyses (e.g., Jochum et al., 2019). For ES sample numbers please see Table 2 in the main text.

ES19C08_008_01_1 *G. ruber albus*

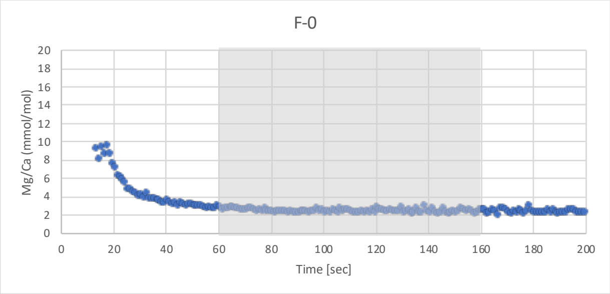

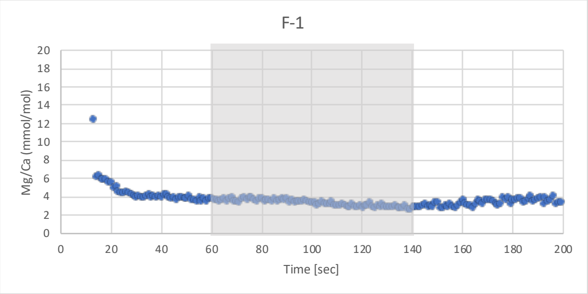

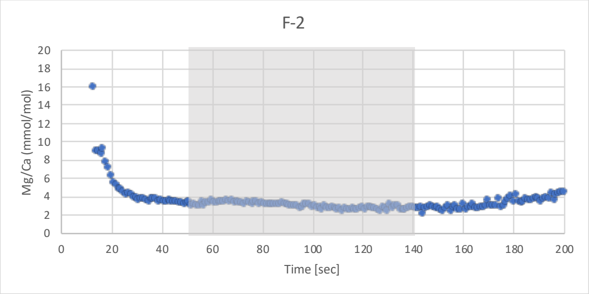

Mg/Ca (mmol/mol): 2.44±0.02 interval averaged: 60-160s

Mg/Ca (mmol/mol): 3.25±0.04 interval averaged: 60-140s

Mg/Ca (mmol/mol): 2.98±0.03 interval averaged: 50-140s

ES19C08_008_01_2 *G. ruber albus*

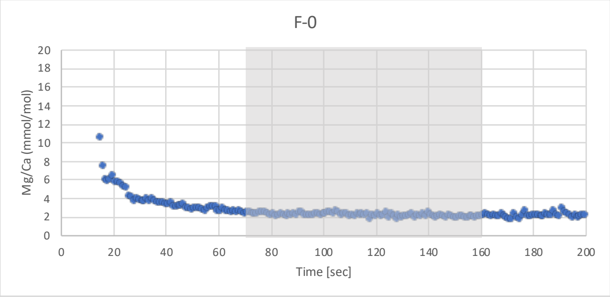

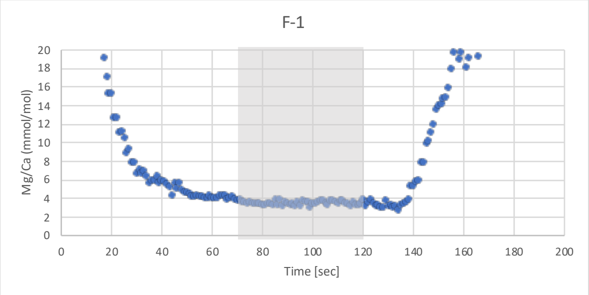

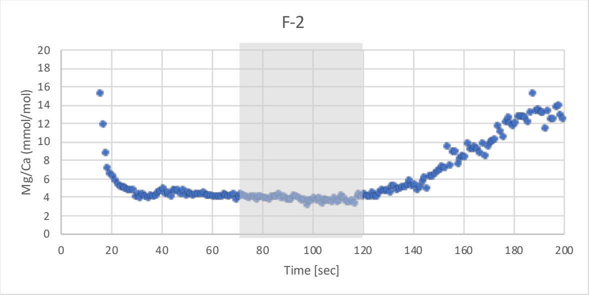

Mg/Ca (mmol/mol): 2.18±0.02 interval averaged: 70-160s

Mg/Ca (mmol/mol): 3.42±0.03 interval averaged: 70-120s

Mg/Ca (mmol/mol): 3.77±0.04 interval averaged: 70-120s

ES19C08_008_01_3 *G. ruber albus*

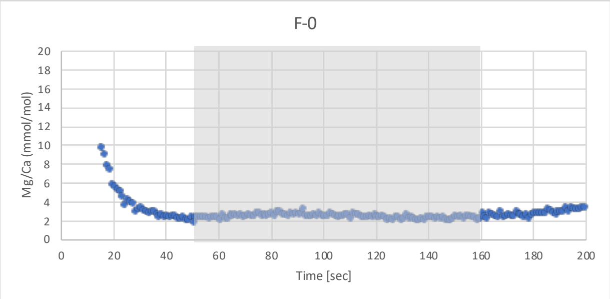

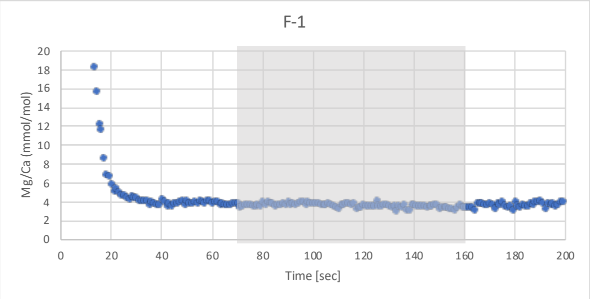

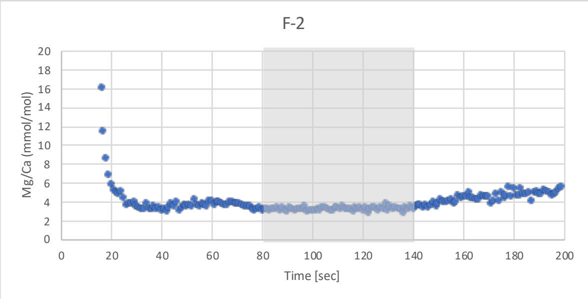

Mg/Ca (mmol/mol): 2.43±0.02 interval averaged: 50-160s

Mg/Ca (mmol/mol): 3.55±0.02 interval averaged: 70-160s

Mg/Ca (mmol/mol): 3.20±0.02 interval averaged: 80-140s

ES19C08_008_01_4 *G. ruber albus*

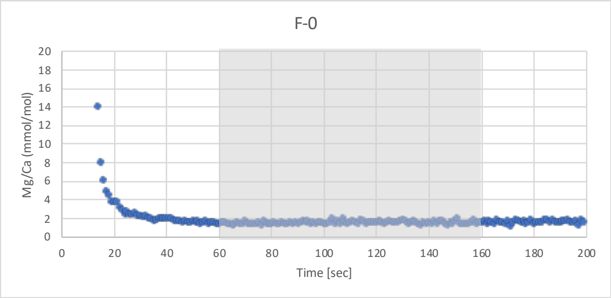

Mg/Ca (mmol/mol): 1.48±0.01 interval averaged: 60-160s

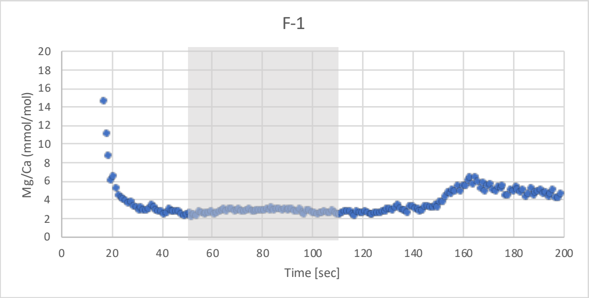

Mg/Ca (mmol/mol): 2.69±0.03 interval averaged: 50-110s

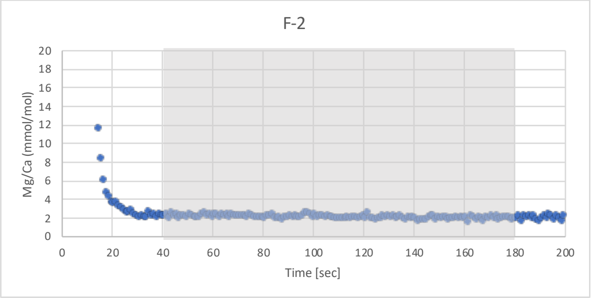

Mg/Ca (mmol/mol): 2.08±0.02 interval averaged: 40-180s

ES19C08_008_02_1 *G. ruber albus*

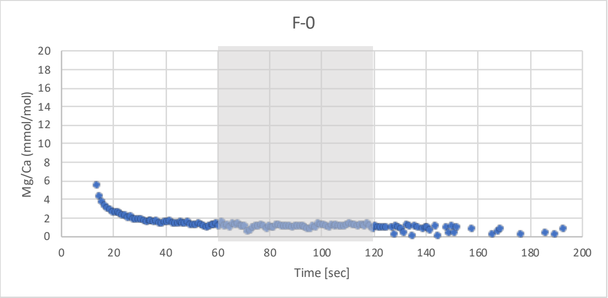

Mg/Ca (mmol/mol): 1.08±0.02 interval averaged: 60-120s

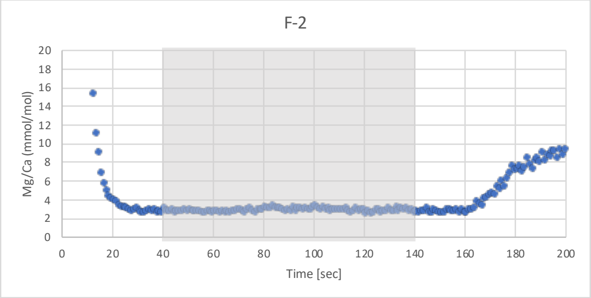

Mg/Ca (mmol/mol): 2.83±0.02 interval averaged: 40-140s

ES19C08_008_02_2 *G. ruber albus*

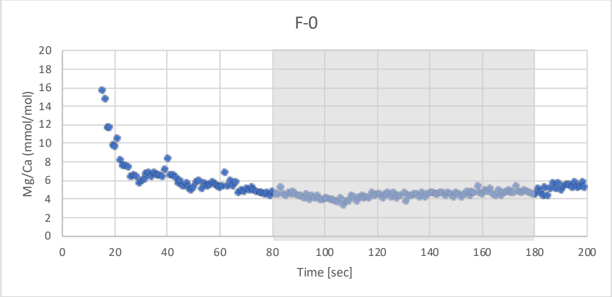

Mg/Ca (mmol/mol): 4.35±0.04 interval averaged: 80-180s

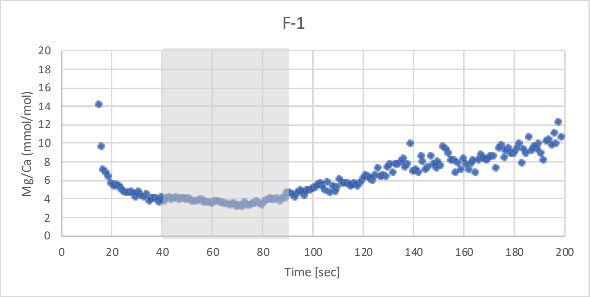

Mg/Ca (mmol/mol): 3.69±0.04 interval averaged: 40-90s

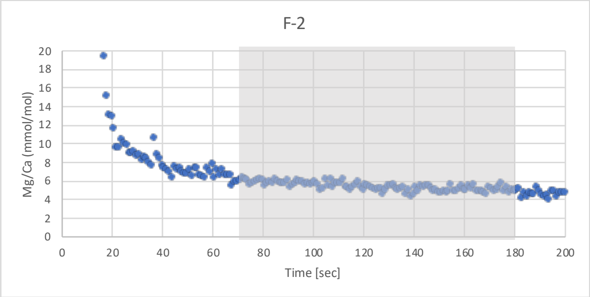

Mg/Ca (mmol/mol): 5.37±0.04 interval averaged: 70-180s

ES19C08_008_02_3 *G. ruber albus*

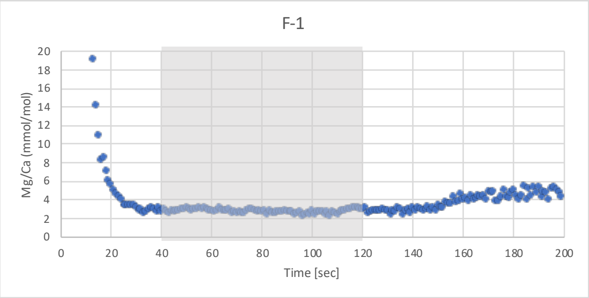

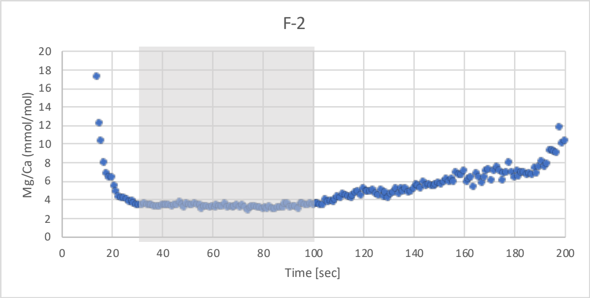

Mg/Ca (mmol/mol): 2.74±0.02 interval averaged: 40-120s

Mg/Ca (mmol/mol): 3.29±0.02 interval averaged: 30-100s

ES19C08_008_02_4 *G. ruber albus*

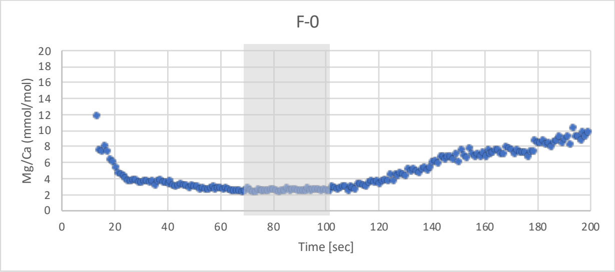

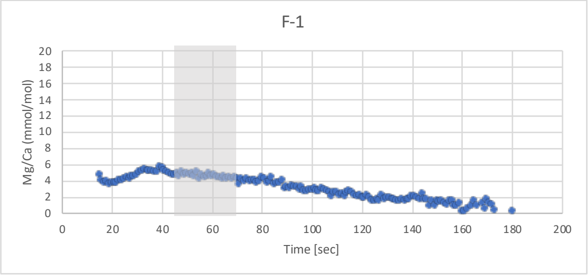

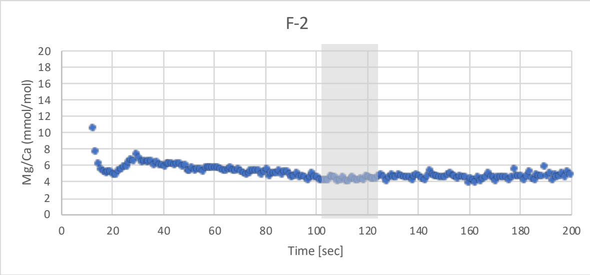

Mg/Ca (mmol/mol): 2.44±0.02 interval averaged: 69-102s

Mg/Ca (mmol/mol): 4.57±0.05 interval averaged: 43-69s

Mg/Ca (mmol/mol): 4.30±0.04 interval averaged: 102-124s

ES19C08_008_02_5 *G. ruber albus*

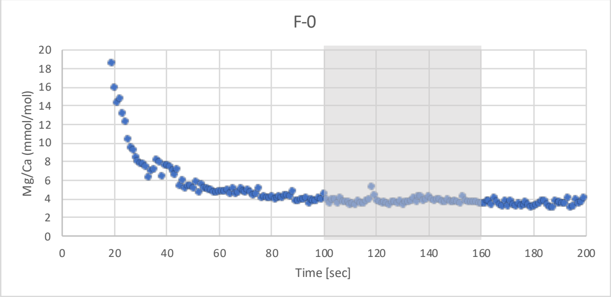

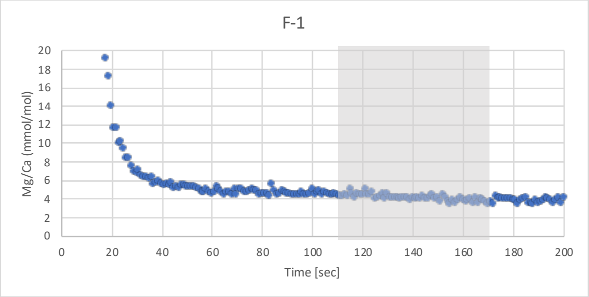

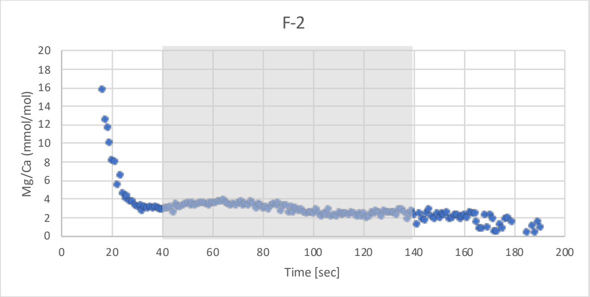

Mg/Ca (mmol/mol): 3.69±0.04 interval averaged: 100-160s

Mg/Ca (mmol/mol): 4.11±0.04 interval averaged: 110-170s

Mg/Ca (mmol/mol): 2.87±0.05 interval averaged: 40-140s

ES19C08_008_02_5 *G. ruber ruber*

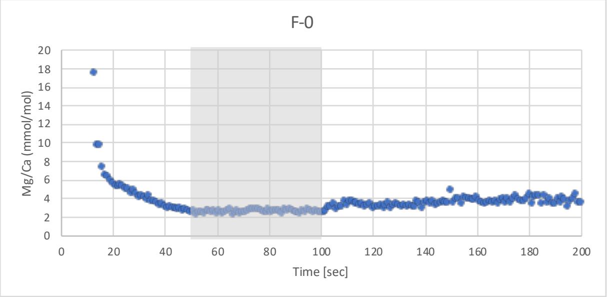

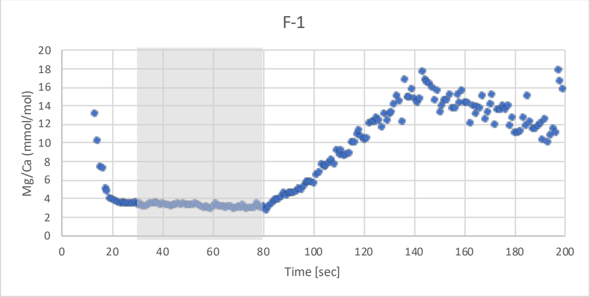

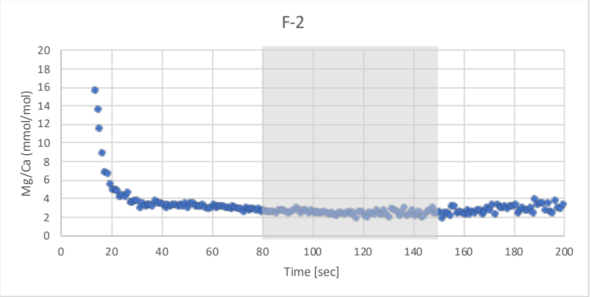

Mg/Ca (mmol/mol): 2.59±0.02 interval averaged: 50-100s

Mg/Ca (mmol/mol): 3.19±0.03 interval averaged: 30-80s

Mg/Ca (mmol/mol): 2.42±0.03 interval averaged: 80-150s

ES19C12_012 _03_1 *G. ruber albus*

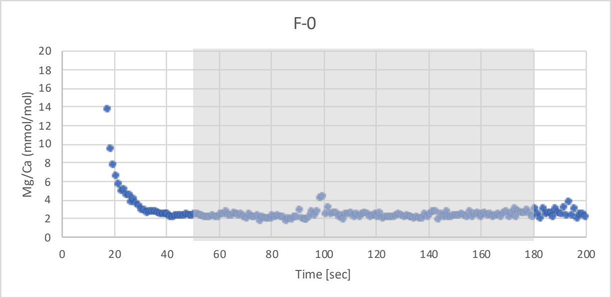

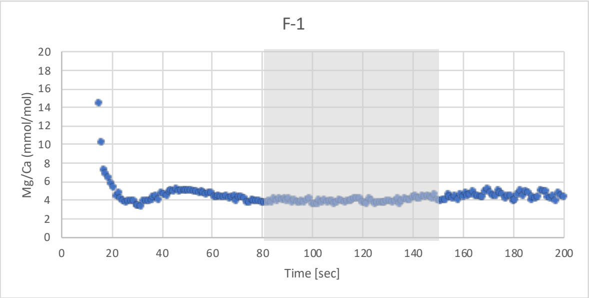

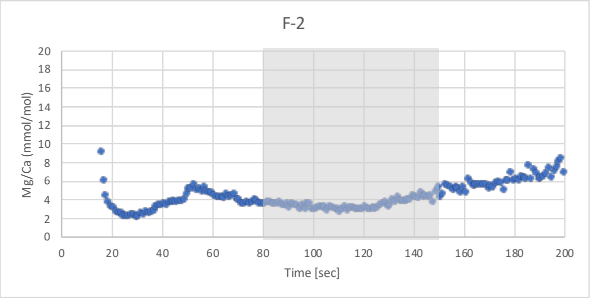

Mg/Ca (mmol/mol): 2.29±0.03 interval averaged: 50-180s

Mg/Ca (mmol/mol): 3.89±0.03 interval averaged: 80-150s

Mg/Ca (mmol/mol): 3.49±0.06 interval averaged: 80-150s

ES19C12_012_03_2 *G. ruber albus*

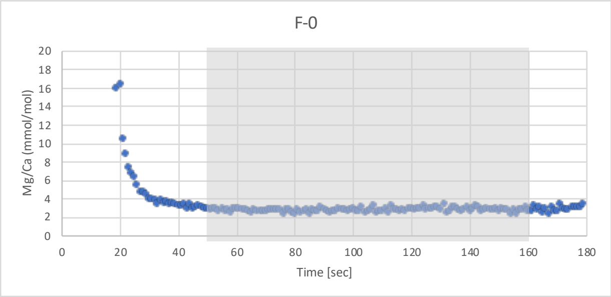

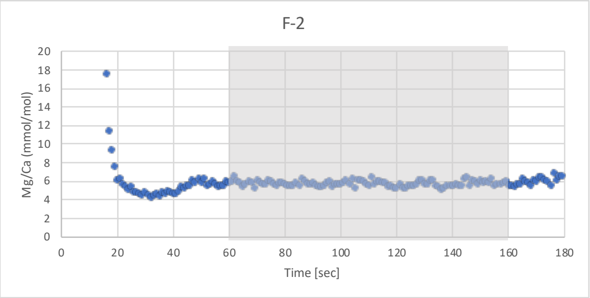

Mg/Ca (mmol/mol): 2.76±0.02 interval averaged: 50-160s

Mg/Ca (mmol/mol): 5.66±0.03 interval averaged: 60-160s

ES19C12_012_05_1 *G. ruber albus*

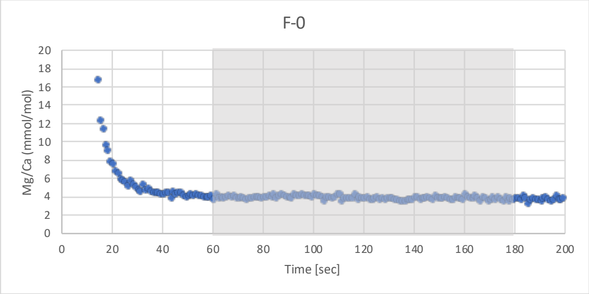

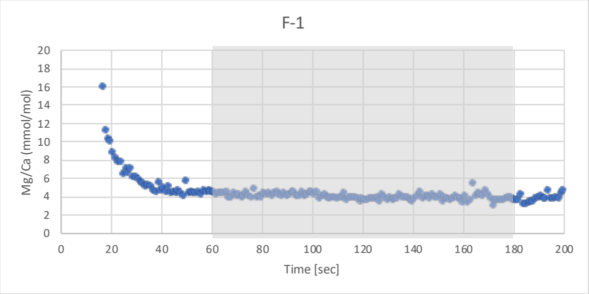

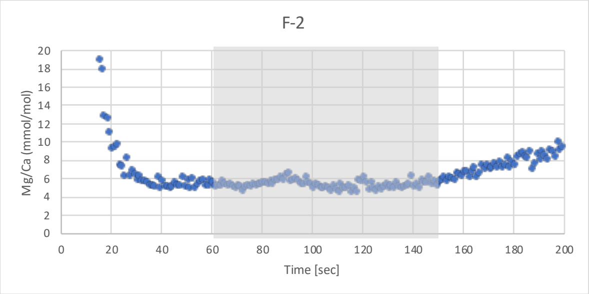

Mg/Ca (mmol/mol): 3.79±0.02 interval averaged: 60 - 180s

Mg/Ca (mmol/mol): 3.97±0.03 interval averaged: 60-180s

Mg/Ca (mmol/mol): 5.31±0.04 interval averaged: 60-150 s

ES19C12_012_05_2 *G. ruber albus*

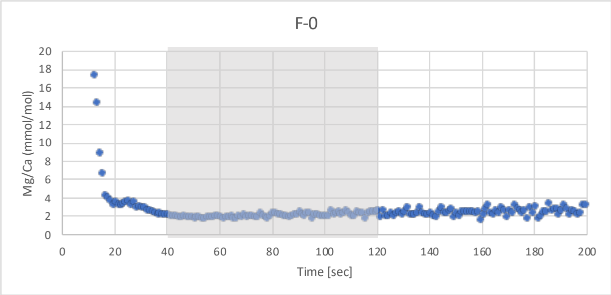

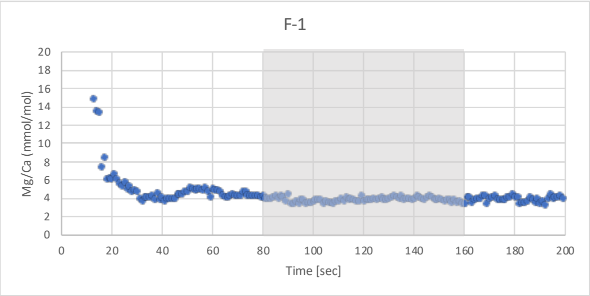

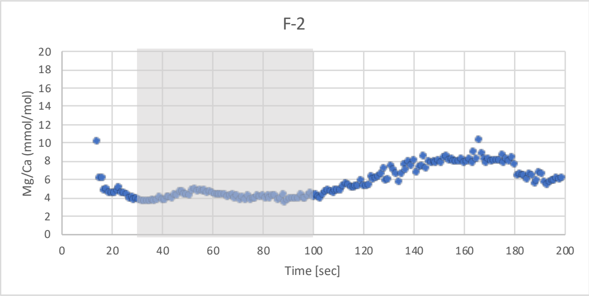

Mg/Ca (mmol/mol): 2.03±0.03 interval averaged: 40-120s

Mg/Ca (mmol/mol): 3.77±0.03 interval averaged: 80-160s

Mg/Ca (mmol/mol): 4.09±0.04 interval averaged: 30-100s

ES19C12_012_06_1 *G. ruber ruber*

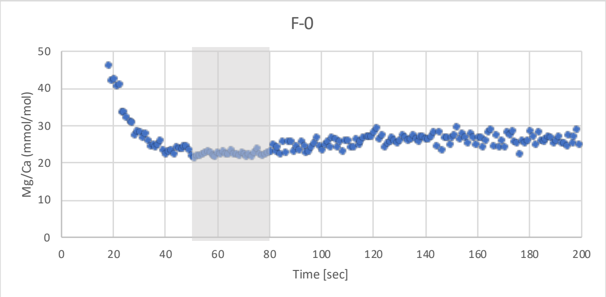

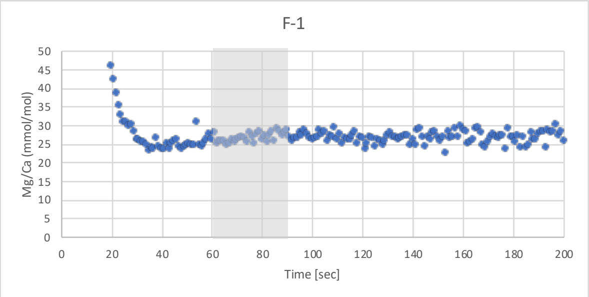

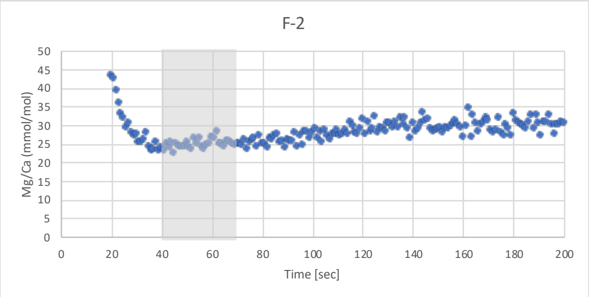

Mg/Ca (mmol/mol): 22.2±0.1 interval averaged: 50-80s

Mg/Ca (mmol/mol): 26.6±0.2 interval averaged: 60-90s

Mg/Ca (mmol/mol): 24.9±0.2 interval averaged: 40-70s

ES19C12_012_06_2 *G. ruber albus*

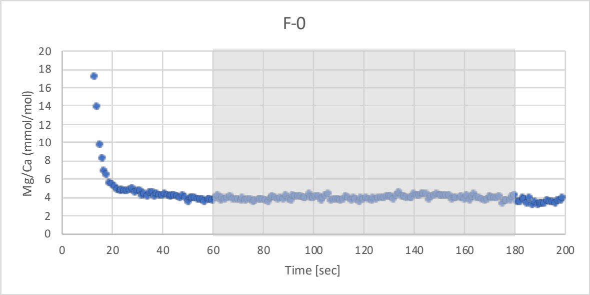

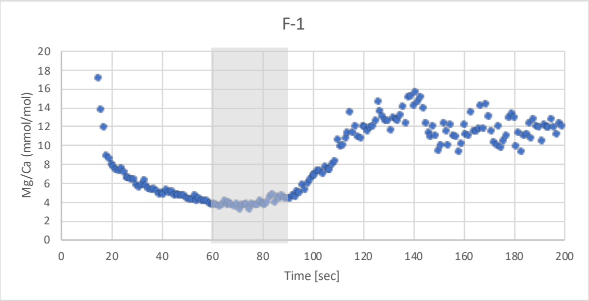

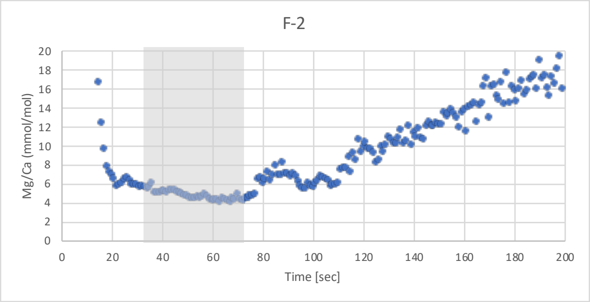

Mg/Ca (mmol/mol): 3.88±0.02 interval averaged: 60-180s

Mg/Ca (mmol/mol): 3.86±0.07 interval averaged: 60-90s

Mg/Ca (mmol/mol): 4.71±0.07 interval averaged: 35-75s

ES19C12_012_10_1 *G. ruber albus*

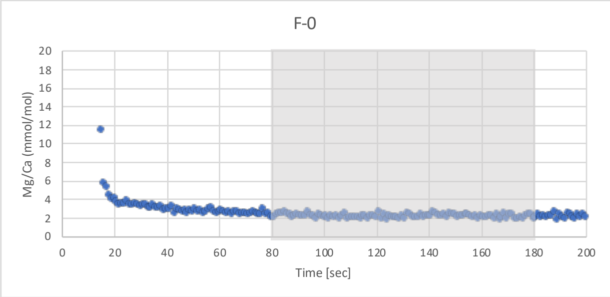

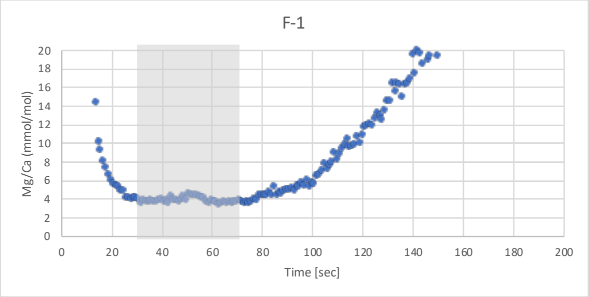

Mg/Ca (mmol/mol): 2.21±0.02 interval averaged: 80-180s

Mg/Ca (mmol/mol): 3.85±0.04 interval averaged: 30-70s

ES19C12_012_10_2 *G. ruber albus*

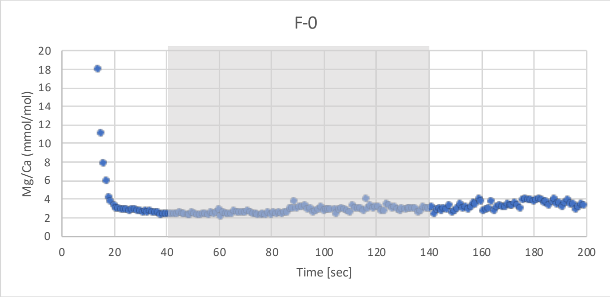

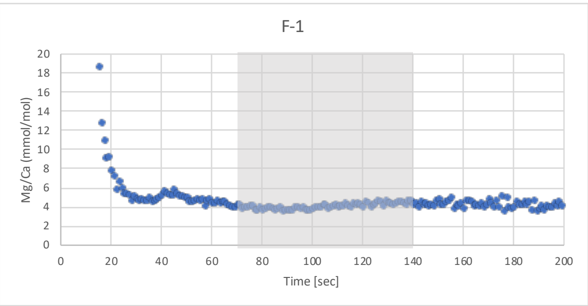

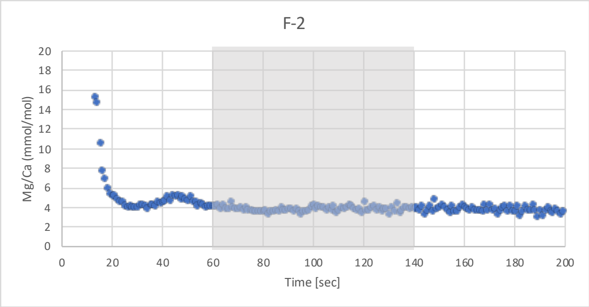

Mg/Ca (mmol/mol): 2.69±0.03 interval averaged: 40-140s

Mg/Ca (mmol/mol): 3.97±0.03 interval averaged: 70-140s

Mg/Ca (mmol/mol): 3.76±0.03 interval averaged: 60-140s

ES19C12_012_10_5 *G. ruber albus*

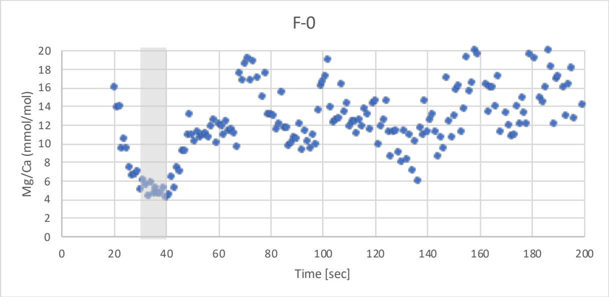

Mg/Ca (mmol/mol): 5.00±0.20 interval averaged: 30-40s

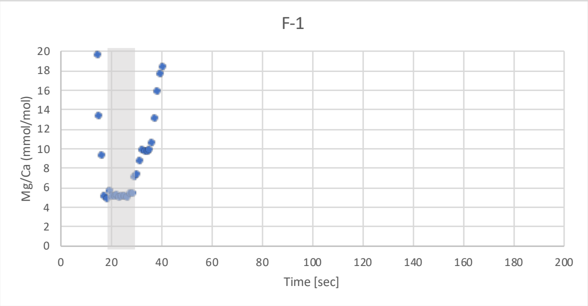

Mg/Ca (mmol/mol): 5.11±0.07 interval averaged: 20-29s

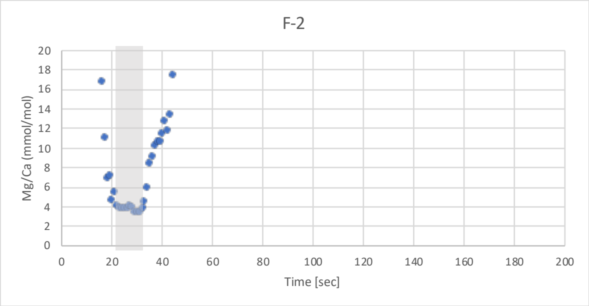

Mg/Ca (mmol/mol): 3.72±0.08 interval averaged: 22-32s

ES19C14_015_03_1 *G. ruber albus*

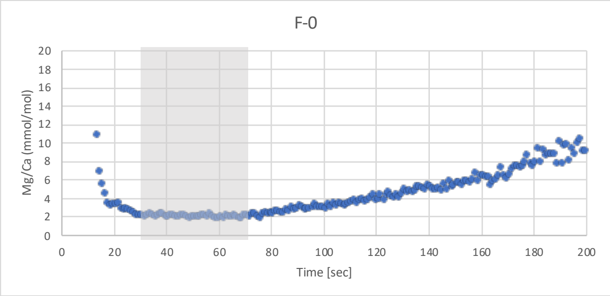

Mg/Ca (mmol/mol): 2.03±0.02 interval averaged: 30-70s

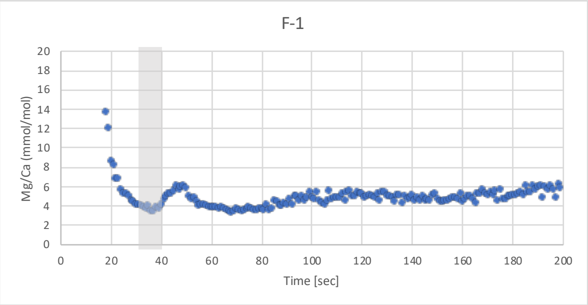

Mg/Ca (mmol/mol): 3.79±0.07 interval averaged: 30-40s

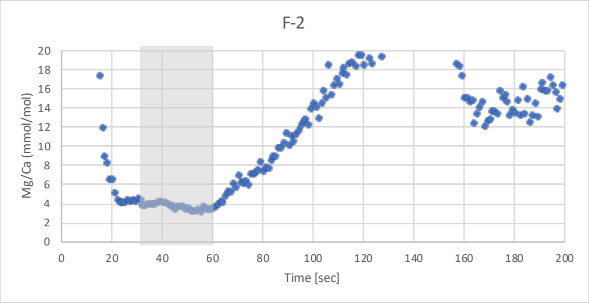

Mg/Ca (mmol/mol): 3.59±0.06 interval averaged: 32-60s

ES19C14_015_03_2 *G. ruber albus*

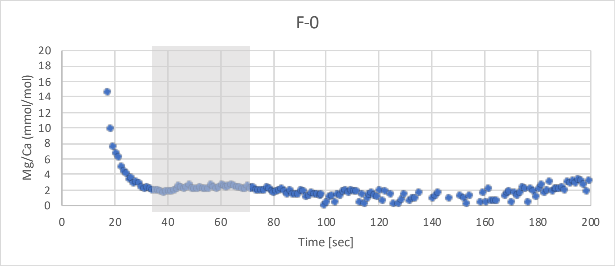

Mg/Ca (mmol/mol): 2.13±0.04 interval averaged: 36-71s

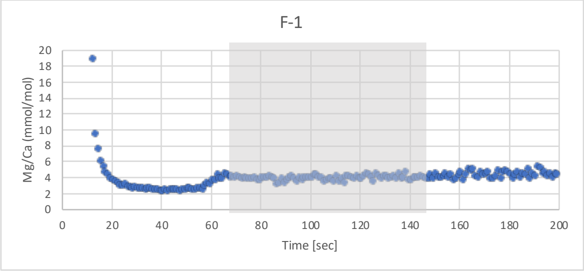

Mg/Ca (mmol/mol): 3.88±0.03 interval averaged: 67-144s

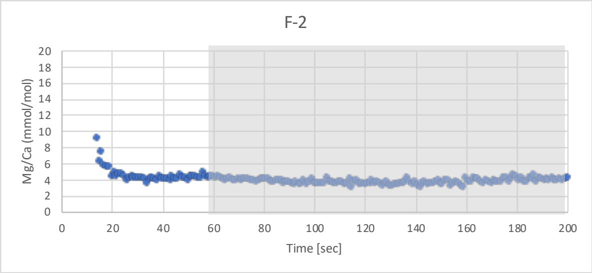

Mg/Ca (mmol/mol): 4.57±0.03 interval averaged: 58-199s
